# ADAM33′s Role in Asthma Pathogenesis: An Overview

**DOI:** 10.3390/ijms25042318

**Published:** 2024-02-15

**Authors:** Jakub Sleziak, Antoni Gawor, Marta Błażejewska, Katarzyna Antosz, Krzysztof Gomułka

**Affiliations:** 1 Student Research Group of Internal Medicine and Allergology, Wroclaw Medical University, 50-367 Wroclaw, Poland; jakub.sleziak@student.umw.edu.pl (J.S.); antoni.gawor@student.umw.edu.pl (A.G.); marta.blazejewska@student.umw.edu.pl (M.B.);; 2 Department of Internal Medicine, Pneumology and Allergology, Wroclaw Medical University, 50-367 Wroclaw, Poland

**Keywords:** ADAM33, asthma, a disintegrin and metalloprotease 33, airway remodeling, polymorphism

## Abstract

Asthma is a complex chronic respiratory disease characterized by airway hyperresponsiveness, inflammation, and obstruction. Many genes have been identified as associated with asthma but none with such substantial significance as the ADAM33 gene due to its role in airway remodeling and bronchial hyperresponsiveness. This review summarizes the current knowledge on the genetic and functional aspects of ADAM33 in asthma pathogenesis. We highlight its genetic variants associated with asthma susceptibility and severity, as well as the functional effects of ADAM33 on airway remodeling, smooth muscle cell proliferation, and its interplay with environmental factors. Additionally, we discuss the potential clinical implications of ADAM33 as a therapeutic target for asthma management.

## 1. Introduction

Asthma is a chronic inflammatory disease involving reversible airway obstruction, bronchial hyperreactivity (BHR), mucus overproduction, and airway narrowing and remodeling. The symptoms include coughing, wheezing, and breathlessness and may become aggravated at night or during physical activity, resulting in decreased life quality, impaired productivity, and significant utilization of healthcare resources. Younger individuals are affected more frequently than adults: 9.1% of children, 11.0% of adolescents, and 6.6% of adults experience asthma symptoms, and the prevalence across all age groups is increased in high-income countries [1]. The worldwide mortality rate for childhood asthma reaches up to 0.7 deaths per 100,000, and children who suffer from severe asthma have a greater risk of developing chronic obstructive pulmonary disease (COPD) in adulthood [2]. Genes play a larger role in children’s asthma [2]. The exact pathogenesis of asthma is still unknown. People from different genetic backgrounds have diverse susceptibilities to asthma; therefore, molecular genetic factors are crucial. The need for determining such genetic factors is still unsatisfied. Early research on genetics in relation to asthma primarily utilized positional cloning along with linkage analysis, which helped identify genes that are associated with asthma and expressed in the airways [3]. The identification of the first such gene coding a disintegrin and metalloproteinase 33 (ADAM33), a risk factor in allergic lung inflammation [4], provided valuable insight. So far, numerous studies have identified ADAM33 as a susceptibility gene for asthma and bronchial hyperresponsiveness (BHR) among multiple populations [5].

This review aimed to gather, assess, and summarize current knowledge on ADAM33′s role in the pathogenesis, development, course, and severity of asthma as well as its possible role in the diagnosis and treatment of this disease.

## 2. Methodology

**Figure 1:**
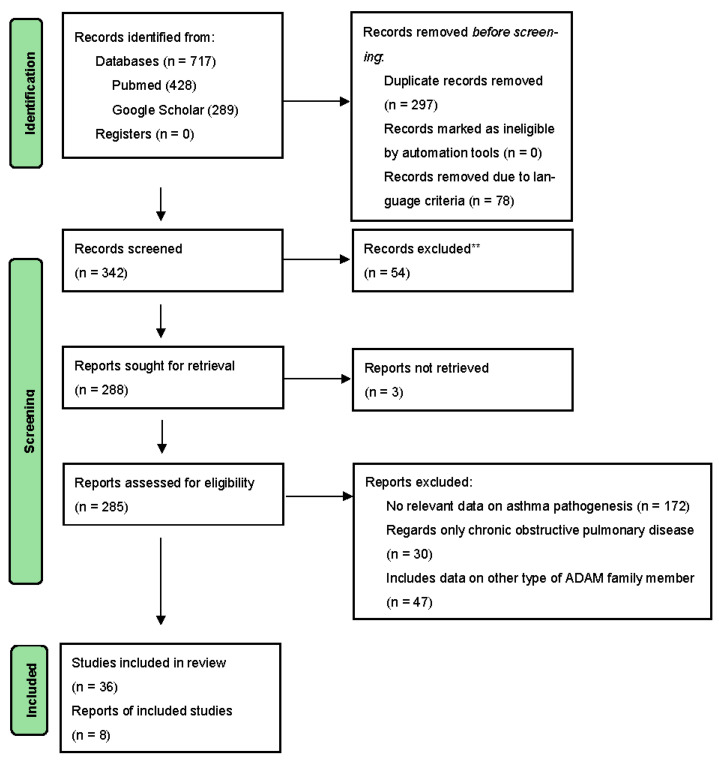
PRISMA flow diagram.

Electronic database searches focused on original articles, meta-analyses, and systematic reviews regarding ADAM33 and its role in the risk, development, course, and severity of asthma. The search process was carried out in PubMed and Google Scholar and comprised a period from 2013 to 21 December 2023. The keywords included “ADAM33”, “Asthma”, “A Disintegrin And Metalloprotease 33”, “ADAM33 and airway remodeling”, and “ADAM33 polymorphism” and were used both in combination and separately. Moreover, articles cited in obtained publications were analyzed and included. Articles in languages other than English were excluded. The search results with the number of records identified, included, and excluded as well as the reasons for exclusions were presented on the Preferred Reporting Items for Systematic Reviews and Meta-Analyses (PRISMA) flow diagram. [Figure 1].

### 2.1. ADAM Molecules in General

**Figure 2:**
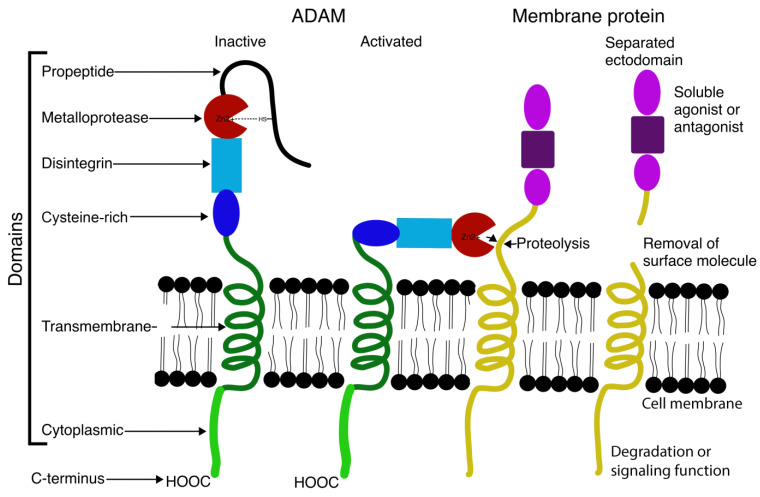
Diagram of ADAM domains and function.

ADAMs are types of zinc-dependent metalloproteinases capable of cleaving peptide bonds of other proteins. These membrane-anchored proteins are structurally related to snake venom disintegrins and contain two functional domains—a disintegrin and a metalloproteinase. They take part in many biological processes including the cell–cell and cell–matrix interactions, such as fertilization, neurogenesis, and muscle development [6,7]. ADAMs’ substrates are typically transmembrane surface proteins, whose cleavage results in the shedding of the ectodomain and the creation of a cell-associated fragment consisting of the transmembrane and cytoplasmic domain [4]. The substrate recognition seems to be dependent on structure-specific qualities with specificity to the substrate-binding pocket in the active site rather than a defined amino acid motif [4]. Other domains such as the cysteine-rich domain, juxtamembrane region, and transmembrane domain are likely needed for proper substrate recognition by ADAM [4]. The EGF domain in other members of the ADAM family was linked with the formation of syncytium that promotes both the thickening and stability of smooth muscle bundles [8] [Figure 2].

**Figure 3:**
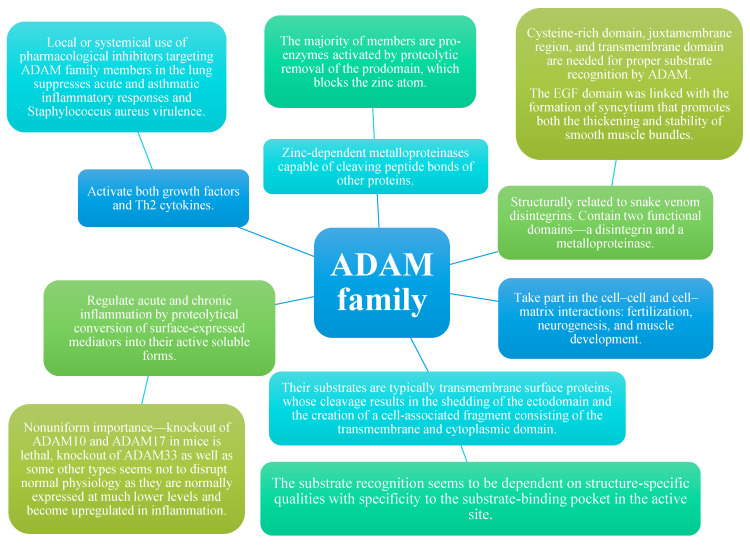
Properties of members of ADAM family [4,6,7,8,9].

ADAMs can regulate acute and chronic inflammation by proteolytical conversion of surface-expressed mediators into their active soluble forms [4]. They can activate both growth factors and Th2 cytokines [9]. Acute and asthmatic inflammatory responses, as well as Staphylococcus aureus virulence, are suppressed with the usage of pharmacological inhibitors that target ADAM family members in the lung either locally or systemically [4]. The majority of ADAMs are proenzymes activated by proteolytic removal of the prodomain, which blocks the zinc atom [4]. The importance of ADAM molecules is not uniform. Although knockout of ADAM10 and ADAM17 in mice is lethal, knockout of ADAM33 as well as some other types seems not to disrupt normal physiology as they are normally expressed at much lower levels and become upregulated in inflammation [4] [Figure 3].

ADAM33 is widely recognized to be a susceptibility gene for asthma with a possible role in the origins of this disease [5,9]. The findings of numerous studies carried out since its discovery turned out to be promising, however, partially contradictory among different or even the same races and populations [10]. It is worth saying that ADAM33 alleles were associated with an increased risk of fibrosis, COPD, BHR, accelerated lung function decline across the life course, and impaired early life function [4,5,11], which emphasizes its importance in lung disease pathogenesis and airway remodeling.

### 2.2. Structure

ADAM33 is a type I 120 kD transmembrane protein [6,12]. Its gene is located on 20p13 and comprises 21 introns and 22 exons encoding a protein of 813 amino acids [13]. ADAM33 consists of a signal sequence, the NH2-terminal prodomain, and catalytic, metalloprotease-like, disintegrin-like, cysteine-rich, epidermal growth factor-like, transmembrane, and cytoplasmic domains [7], with an mRNA sequence that borders with the 3′-untranslated region (UTR) [9]. A zinc atom bound in the active site is crucial for its proteolytic ability [4].

Northern blot analysis revealed two transcripts of ADAM33: one band at 5.0 kb and a second of lower intensity at 3.5 kb. Northern blot analysis of total cellular and cytoplasmic RNA revealed that the larger transcript was not present in the cytoplasm of bronchial smooth muscle cells, suggesting the occurrence of a splicing process and that the larger message may contain unspliced intronic regions [14]. Regulatory elements located within those regions contain several important SNPs, and it has been suggested that they may control the efficiency and kinetics of splicing [15]. So far, a complex variety of splicing pathways have been described, such as deletion of exon Q, partial deletion of exon R, deletion of whole or of parts of the PRO/MP domains, and/or deletion of other domains (e.g., secretion signal or disintegrin domain) [15]. Alternative spliced forms of ADAM33 were identified in lung-derived cDNA, and one of the variants is predicted to be a soluble form; moreover, it has been implied that there are other ADAM33 species of yet unknown composition [8].

### 2.3. Function

ADAM33 is found mainly on cell membranes in airway smooth muscle cells (SMC) and myofibroblasts [7]. It has the ability to regulate angiogenesis, cell differentiation, and proliferation [7]. With its physiologically balanced activity, it plays a protective [16] and regulatory role [8] since it can release growth factors and modify cell surface receptor expression [8]. ADAM33 was found to positively affect cell fusion [13]. Furthermore, its action significantly impacts the activity of adhesion molecules and cytokines through the release of soluble ectodomains, resulting in either loss of function of a cell surface molecule or transduction of an intracellular signal [4]. It impacts proliferation, differentiation, signaling, and apoptosis [17]. Another role of ADAM33 is to keep cell connections intact, thus protecting cells from the penetration of injurious agents [18]. While ADAM33 was previously thought to be expressed only in mesenchymal cells, recent studies have indicated that it may also be expressed in the epithelial cells of patients with severe asthma [4,19]. Interestingly, high levels of ADAM33 mRNA were observed in the granulation tissue of a duodenal ulcer, which indicates ADAM33’s possible role in repairing injured tissues [8]. In addition, it was also found in lung leukocytes, the endothelium, and the basal layer of the epithelium [4]. Research has linked ADAM33 to airway remodeling and BHR through the epithelial–mesenchymal trophic unit (EMTU), leading to the proliferation of biosynthetically active fibroblasts, myofibroblasts, and smooth muscles [3]. A loss-of-function mutation of the ADAM33 gene could result in impaired shedding of receptors of growth factors, resulting in increased cellular proliferation. Conversely, a mutation resulting in a gain of function could result in an increase in the shedding of growth factors as well as pro-inflammatory cytokines, causing the type 2 helper lymphocyte-mediated immune response [16]. Indeed, it is suggested that it takes part in the activation of Th2 cytokines [13].

Despite ADAM33 being a membrane protein, its soluble form (sADAM33, 55 kD) is found in high levels in asthmatic airways, which correlates with reduced lung function [5]. A substantial interaction between sADAM33-mediated airway remodeling and sensitivity to allergen exposure, leading to allergic inflammation and BHR in early life, has been found [5]. sADAM33 cleaves membrane domains of stem cell factor c-kit, TNF-related activation-induced cytokines, insulin beta chains, and amyloid precursor proteins [4]. It promotes angiogenesis and myogenesis in vitro [5,12] and therefore further impacts airway remodeling independently of inflammation, and its high levels were linked to reduced lung function and the severity of asthma [5,9].

A part of ADAM33′s gene containing a single-nucleotide polymorphism (SNP) T_2 encodes the cytoplasmic tail. T_2 allele A, which is known to be associated with asthma exacerbations in adult patients with type 2 inflammatory endotype, may determine vulnerability to shedding and turning into sADAM33 [20]. sADAM33′s release is stimulated by transforming growth factor-β2 (TGFβ2) [20]. Maternal allergy during pregnancy triggers the increase in sADAM33 protein and implies the following increase in airway and vascular smooth muscles in utero; however, those changes elicit neither inflammation nor BHR in transgenic mice [5]. Similar changes were observed after the exposure of human embryonic lung explants to sADAM33 in vitro [5]. In the transgenic mice, the remodeling changes induced by sADAM33 did not cause BHR in response to inhaled methacholine and did not increase inflammation [5]. While on its own this remodeling did not impact either BHR or inflammation, the same study proved that the expression of inflammation and remodeling genes, as well as mucous production, was enhanced by sADAM33-expressing mice only after exposure to a common human aeroallergen, house dust mite (HDM) extract. That result was followed by an increase in airway resistance and bronchoalveolar lavage fluid (BALF) eosinophilia [5]. This indicates that sADAM33 increases susceptibility to the Th2 stimulus. The reason behind this could be that the sADAM33-induced airway remodeling causes an increase in the population of fibroblast and smooth muscle cells that are able to produce mediators that intensify inflammatory response [21,22]. In addition, the increased angiogenesis caused by sADAM33 could facilitate the migration of eosinophils to lung tissues.

In the other murine model of chronic airway inflammation induced by allergens, there was an observed upregulation of ADAM33 mRNA expression in the lungs [23], and elevated levels of both ADAM33 mRNA and protein were identified in the BALF and bronchial biopsies obtained from individuals diagnosed with asthma [24].

ADAM33’s expression is upregulated during both acute and chronic lung inflammation in patients with COPD and sarcoidosis [4]. Tissue immunoexpression of ADAM33 was higher in viral pneumonia-positive patients than in virus-negative cases [18]. This suggests that ADAM33 plays an important role in the severity and chronicity of the inflammatory process of the lungs and its consequences. Individuals with asthma tend to have an increased expression and enzymatic activity of ADAM33, and this correlates with asthma severity and lung function deterioration, measured in FEV1% (the percentage of the forced expiratory volume in the first second, relative to the total amount of air exhaled during the entire forced vital capacity (FVC) maneuver) [5,13,25,26,27]. However, the findings of research on the ADAM33–asthma severity relationship are partly inconsistent since certain investigations on this topic showed no significant association [28].

ADAM33’s increased expression has a potential role in asthma’s eosinophilic/type 2 inflammatory endotype, known to be the most severe and treatment-resistant, which requires the development of novel and nonsteroidal therapeutics [20]. Furthermore, ADAM33 was reported to be associated with a mixed type of eosinophilic/type 2 and neutrophilic inflammations [20]. Its expression was proven by studies to be increased in human fibroblasts in vitro and in asthmatic patients by exposure to interleukin 4 (IL-4) and IL-13 in a time- and concentration-dependent manner [20,23]. In mice, its expression was increased also after stimulation with IL-33 [4]. Interestingly, the response to exposure to HDM extract was substantially different in ADAM33 knockout mice in comparison to the wild-type mice. In the knockout mice, the expression of Th2-type inflammatory genes Ccl11, Il-5, and Il-13, but not Cxcl1, was suppressed and CCL11, IL-5, and eosinophil levels were reduced in BALF, which indicates ADAM33′s role in the regulation of Th2-type inflammation [5].

Research on epigenetic regulation of ADAM33 showed no difference in the methylation pattern of its promotor between asthmatic and regular fibroblasts. [29]. However, it proved the regulatory hypermethylation of CpG islands within its promotor in the presence of epithelial cells and the corresponding hypomethylation in fibroblasts; therefore, this mechanism probably impacts cell-specific expression. [29]. The repression of ADAM33 in this mechanism appears to be a consistent characteristic of airway epithelial cells, regardless of the presence or absence of asthma [19]. On the other hand, significant differences in methylation patterns in ADAM33 were found between healthy individuals and patients with allergic rhinitis, and hypermethylation of ADAM33 was significantly associated with lower eosinophil counts [30]. TGF-*β*2 was found to contribute to chromatin condensation with deacetylation of histone H3, which downregulates ADAM33 expression in both normal and asthmatic fibroblasts [7]. An increase in TGF-*β*1 levels in the airways of asthmatic patients both suppresses ADAM33 and promotes its external domain shedding [7].

### 2.4. SNPs

SNPs are the most frequent type of genetic variation found among individuals, and they are strongly linked to susceptibilities to many diseases [2]. The University of California Santa Cruz Genome Browser indicates 90 SNPs observed within the ADAM33 gene [31]; nevertheless, the literature indicates the total number of these SNPs exceed 340, with 35 indicated in the literature as possibly associated with asthma [32].

Since the discovery of ADAM33, numerous SNPs have been assessed for association with asthma among various populations, and even though such association was identified for at least one SNP in each population, none of the SNPs could be associated with asthma phenotype across all populations, which may be caused by compound effects of multiple alleles, genes, and environmental factors; the difference in exposure to allergens in separate geographical regions; or linkage disequilibrium patterns between SNPs and the undetected causative defect in gene [7]. Studies have indicated that multiple SNPs may work simultaneously to raise the risk of developing asthma [2]. The correlation between SNPs and asthma is influenced by various factors including region, ethnicity, age, and sex [2]. So far, more than 100 SNPs have been reported to be associated with asthma [9]. It was demonstrated that SNPs in ADAM33 are associated with lower lung function in young children and a faster decline in lung function in individuals with asthma and COPD, as well as in healthy individuals [7,19]. True causative variants of ADAM33 polymorphisms in asthma development have not yet been ultimately identified. Below, we discuss the findings of research on the SNPs most frequently mentioned in publications.

In a recent comprehensive meta-analysis comprising 63 articles, with 13,280 asthma patients and 13,340 controls, T2, Q1, and F + 1 polymorphisms were associated with asthma risk in the Asian population; V4 polymorphism was related to asthma risk in the Caucasian population; T2, F + 1, and ST + 4 polymorphisms were associated with asthma risk in children; and Q1 along with V4 polymorphisms were associated with asthma risk in adults. [9] It is worth saying that despite the relatively large group of analyzed patients, its results were either inconsistent or only partly consistent with other previous meta-analyses [13,33,34,35]. Notably, the correlation between F + 1 and asthma has been observed in multiple populations, such as Jordanian, Chinese, Northern Indian, German, and Icelandic populations [36], and this association was particularly strong in the Asian population [37]. The same SNP was associated with altered lung function in early life (3 and 5 years) [36].

In a large meta-analysis, ADAM33 V4 polymorphism was associated with an increased risk of asthma with no significant differences in population selectivity. The authors indicated this polymorphism as a biomarker that could be utilized for early diagnosis of asthma [13]. V4 is located in the 3′UTR of the gene, whose changes may affect transcription, and since it is located in proximity to T1, it was suggested that their relationship can impact the development of asthma [36,37].

A previous meta-analysis found that in both the total population and Chinese individuals, there was no observed association between V4 and the likelihood of developing pediatric asthma [2]. On the contrary, another meta-analysis shows a strong association between the V4 genotype and incidence of asthma in Jordanian children in both codominant and recessive models as well as in children from the United States/United Kingdom combined population, the United Kingdom only, Dutch White, and Indian populations [36].

T1 polymorphism was proven to have a significant association with asthma risk and impaired lung function in Asian children, with increased inflammatory cell counts, and with a decline in lung function in patients with COPD [34]. The T1 site corresponds to the domain involved in intracellular signaling [34]. The presence of the A allele of ADAM33 T1 in individuals with asthma was found to be associated with higher eosinophil count and increased airway hyperresponsiveness compared to the presence of the C allele [38]. Research from the Netherlands showed that S1 and QK 1 SNPs of ADAM33 were significantly associated with a progressive decline in FEV1 in asthmatic patients over a 20-year period [5].

An observational study on asthma exacerbations in 217 Asian patients did not reveal any genetic risks for exacerbations [20]. However, after including the effect of the type 2 endotype, a significant positive interaction was observed between the type 2 endotype (serum periostin level of ≥95 ng/mL) and A allele of the ADAM33 T2 SNP; therefore, this allele was found to be a significant risk marker of subsequent exacerbations in patients with type 2 endotype but other examined variants of ADAM33 S2, T1, and V4 showed no such association [20].

In the Chinese Han population, a significant correlation was found between Q-1 [9] and asthma risk, while in Caucasians, it was associated with asthma in children [14]. Individuals who were homozygous for minor alleles of SNP Q-1 (CC) experienced a rapid decline in FEV1 of 9.6 mL/year [36]. The Q-1 SNP is located in proximity to exons Q, P, and R, which comprise the epidermal growth factor (EGF) domain. This may explain the role of Q-1 SNP in asthma, as it can influence splicing and therefore affect EGF signaling, which is crucial for lung morphogenesis [36].

The S2 variant has been linked to asthma in several populations, including Jordanian, British, Europeans, Black Americans, White Americans, Hispanic Americans, and Thai individuals, and since it is a silent mutation, the biological explanation for the effect is that it could be in linkage disequilibrium with the true causative SNP [36,37]. According to the results of another meta-analysis, the S2 SNP was not associated with the risk of pediatric asthma development in the total and the Chinese population [2].

The significance of a particular SNP among one race can vary depending on specific subpopulations. A large case–control study showed that although a significant association was found for ST + 7 and no association was observed for S1, S2, ST + 4, and V-1 among both US White and Dutch White populations, the significance for asthma association with V4 was significant only for the Dutch population [10].

More and more studies present the position regarding ADAM33 polymorphisms and their impact on asthma. This year’s publications (e.g., May, November 2023) reinforce the belief that T1, T2, and S1 are factors in asthma. The first mentioned refers to the significance of T1 polymorphic in the Arab population. The second publication also refers to their connection to aeroallergen-induced asthma for the population of West Bengal, India [39,40].

Allergological diseases, such as allergic rhinitis (AR), allergic skin dermatitis, and bronchial asthma, generate oxidative stress. The antioxidant levels next to IgE concentrations play a significant role in such states. There is evidence of a strong correlation between increased IgE levels and decreased antioxidant levels in asthma patient populations with ADAM33 (V4) polymorphism when compared to control patients; however, no particular association was found between IgE levels and V4 polymorphism in both asthmatic and healthy control groups [41].

In light of the ongoing global research on SNPs, there is a need to highlight the recent promising developments in these studies. This year’s study from South India explores the relationship between ADAM33 polymorphisms and treatment responsiveness. The results showed that SNP S + 1 demonstrated a correlation with asthma, the minor allele “T” for S + 1 was connected with mild severity of the disease, and the homozygous minor allele of SNP V4 was identified as linked to decreased lung function and the least improvement in lung function after three months of inhaled corticosteroids (ICS) + long acting beta-adrenoceptor agonist (LABA) therapy [42]. This presented relation could be applied in the future for diagnostic and individual therapy, particularly with V4 polymorphism as a genetic indicator for predicting the response to ICS + LABA therapy within the studied population [42]. The data on aforementioned SNPs are summarized in the table below [Table 1].

### 2.5. ADAM33 Haplotypes’ Association with Asthma

Current investigations extend the examination of singular SNPs within the ADAM33 gene, progressing toward the exploration of haplotype combinations of alleles for different SNPs of a specific gene and their associations with asthma [43]. 

Such analyses are particularly advantageous due to their potential to capture causal variants more accurately than individual SNPs and may themselves be potent variants influencing disease [32,44].

In the study, which examined controls selected from the general population from the Burden of Obstructive Lung Disease cohort from urban Mysuru, India, the SNPs T2 and T1 demonstrated a significant association with asthma, exhibiting strong linkage disequilibrium. These SNPs, though individually benign, in haplotype form reveal a stronger correlation with the disease. The association of haplotype with asthma was analyzed using Haploview software, which uses its own statistical value T-int. A T-int value exceeding 100 is deemed indicative of significant association and was found to be 105.54 in the observed association [32].

The findings corroborated another investigation analyzing six polymorphisms and their haplotypes within ADAM33, examining their correlation with asthma among the population of the Caribbean coast of Colombia. The haplotype GCAGGG in S2/ST + 7/T1/T2/V-1/V4 exhibited a significant linkage with asthma in the familial-based analysis [45].

During the investigation into the association of the ADAM33 gene with asthma and BHR within a combined UK and US population, five pairs of SNPs within ADAM33 demonstrated correlations with asthma at a significance level of *p* < 0.001. These pairs included ST + 4/V-3, ST + 4/V-2, V1/V4, V2/V4, and V4/V5, with the most notable pairs, ST + 4/V-3 and ST + 4/V-2, achieving a *p* value of 0.00004. In the UK sample subset, five pairs of SNPs also exhibited significance at the *p* < 0.001 level, comprising ST + 4/V-3, ST + 4/V-2, S2/ST + 4, ST + 4/ST + 5, and S + 1/ST + 4, with the most remarkable pair being ST + 4/V-3 (*p* = 0.000003) [14].

Investigation in the Australian population proved the haplotypic association with asthma and its severity, driven by V_1 and ST + 7 SNPs with a *p* value of 0.00016 [46].

A robust correlation with asthma was identified in the Japanese population, specifically with the CCTG haplotype in S2/T1/T2/V-3, indicating a statistical association at a *p* value of 0.0024 [47].

In the Uygur population of China, the occurrence rates of the CAC and CAT haplotypes (both in S + 1/T1/F + 1) are notably elevated among individuals diagnosed with asthma when compared to those in healthy control groups, intimating that these specific haplotypes might confer an increased susceptibility to asthma [48].

Raby et al.’s [49] family-based study on childhood asthma and ADAM33 in Caucasians revealed an intriguing association between a common 16-SNP haplotype and the disease.

Contrastingly, the study conducted in the Korean population did not reveal any significant association between certain SNPs (S1, T1, V-1, V4) or their haplotypes and asthma susceptibility [50].

This geographic and ethnic variation in haplotype–disease associations signals the profound effects of genetic diversity across populations, which must be considered to examine the complex genotypic foundations of asthma [45].

## 3. Environment

Environmental chemicals may impact the genetic association with asthma. Moreover, this relationship may vary between various populations. The homozygous mutant genotype of ADAM33′s SNP ST + 5 was found to protect against pollution caused by heavy traffic [7]. Lower methylation levels of ADAM33 promoter were found in children with daily life pet exposure and AR compared to a corresponding control group [30]. The interaction was significantly related to AR risk; however, this study excluded asthmatic patients, and therefore no direct conclusion could be made regarding asthma [30].

Significant interactions have been found between polymorphisms in 20p13-p12 and asthma presence and severity with early-life tobacco smoke exposure [51]. An association of SNP V4 heterozygotes with reduced FEV1 among smokers has been observed [52]. Another study detected an interaction between prenatal exposure to cigarette smoke and the S1 and S2 SNPs in relation to the development of BHR with no significant interaction observed for postnatal exposure. Additionally, the S1 was found to be associated with respiratory impedance at the age of 8, while the S2 was associated with predicted FEV1% [53]. Previous research indicated that early-life tobacco smoke exposure in asthmatic children, who are homozygotes for the G allele in rs512625 (ADAM33), increased the risk of being hospitalized due to asthma exacerbation by 9-fold, whereas there was no influence of early-life tobacco smoke exposure in the rest of the group in that study. Furthermore, children with the same particular SNP and cigarette smoke exposure had lower forced expiratory flows measured at the mid-portion (FEF50%) [52].

These findings suggest that genetic variations in ADAM33, in combination with exposure to cigarette smoke, may influence the development and progression of respiratory diseases. However, further research is necessary to fully understand the complex interplay between genetic and environmental factors and their effects on respiratory health.

In addition to the experience of COVID-19 in recent years, the connection between ADAM33 SNP and the risk of severe disease is determined. During this time, rs7216389 (an SNP in the ORMDL3 gene) and rs2280091 (T1) were described. However, no significant association was found. These two polymorphisms are no longer considered suitable markers in the prediction of severe COVID-19 in the asthma population. Nevertheless, further attempts will be taking place [54].

## 4. Proposed Pathogenesis

Frequent allergic reactions and exposure to harmful chemical substances can cause an increase in TGF*β*1 levels, causing migration of mesenchymal stem cells and resulting in fibrosis. The development of the Th2-mediated immune response upregulates ADAM33, and due to an interaction between genetic predispositions and environmental influence, its expression is impaired, which could lead to a synergic pathological remodeling of the airways.

The allele of ADAM33 T2 may take part in the development of severe asthma by amplifying the mixed type of eosinophilic/type 2 and neutrophilic inflammation (the most severe endotype of asthma) since a significant association was found between this allele and serum IL-6 levels as well as higher blood neutrophil counts, which were emphasized in patients with eosinophilia type 2 inflammatory endotype [20]. Moreover, a significant positive interaction between eosinophilia and the allele was observed and a univariate analysis identified the ADAM33 T2 allele as a risk marker for patients with eosinophilia and subsequent exacerbations [20]. On the other hand, an analysis of an independent cohort of asthmatic patients showed the association between the ADAM33 T2 A allele and higher blood neutrophil counts only in patients with eosinophilia and not in the general group [20]. No allergic asthma phenotype was found in ADAM33 knockout mice (129Sv X C57BL/6) [17]. Nevertheless, it is not clear whether ADAM33 functions in the same way in humans and mice. The ADAM33 gene may interact with other genes in its role in asthma pathogenesis. A combined effect on the risk of developing asthma was observed with the presence of polymorphisms in the interleukin 4, β2-adrenergic receptor, and ADAM33 genes. The T allele for the IL-4 gene promoter (C-589T locus), the C allele frequency for ADRβ2 (Gln27Glu locus), and the A allele for the ADAM33 gene (rs2280091, T1) were higher in asthma patients than in normal individuals. The study found that the risk of developing asthma increased with the presence of these genetic variations, indicating an additive effect, and that their combination increased asthma’s diagnostic sensitivity and specificity [38].

Having taken under consideration ADAM33’s role in asthma development and pathogenesis, its use as a therapeutic target has been suggested. Airway remodeling is a rather irreversible process. However, it was proved that in transgenic mice, in which airway remodeling was previously induced by its exposure to soluble ADAM33 (sADAM33), after 28 days without sADAM33 expression, the airway remodeling was significantly reduced with a visible decline in airway and vascular smooth muscle tissue [5]. This indicates a possibility of a novel asthma treatment directed toward the inhibition of ADAM33′s activity. Historically, targeting metalloproteases for the treatment of diseases like cancer has been challenging due to the occurrence of off-target effects [5]. Nevertheless, distinctive characteristics are present in the substrate-binding site, enabling the development of selective inhibitors for this enzyme via structure-based design [5]. Inhibition of ADAM33 by small-molecule compounds and tissue inhibitors of metalloproteinases (TIMPs) has been already observed. Among the latter group, TIMP-3 and -4 demonstrated a high level of activity, while TIMP-2 showed less activity and TIMP-1 did not exhibit any activity at all, which seems to be distinctive and has not been reported in other members of the ADAM family [8].

When human smooth muscle cells (SMCs) were transfected with miR-708 (which regulates the MAPK and PI3K signaling pathways), it was observed that the expression of ADAM33 genes was inhibited. This indicates that miR-708 may offer a new therapeutic option to decrease airway inflammation and ASM proliferation in individuals with asthma. Despite those findings, no asthma therapy targeting ADAM33 has been so far developed, which indicates the necessity to perform more research on this topic.

Another suggested direction for further research is the relation between ADAM33 and the cadherin-related protein alpha-catenin. Its lower expression leads to increased eosinophil invasion and epithelial denudation due to damage to the airway epithelial barrier [55]. The main causes of asthma exacerbations are epithelial cell abnormalities such as epithelial denudation, ciliary destruction, and growth factor and receptor expression [55]. Since both alpha-catenin and ADAM33 are involved in cell–cell interactions, they likely interact with each other through signaling in the binding between epithelial cells. Therefore, loss of expression of these molecules can lead to epithelial exposure. Unfortunately, this interaction has not been documented in the literature and thus requires investigation.

### 4.1. Conventional Treatment Responsiveness

Given the points raised earlier, it appears necessary to discuss the relationship between ADAM33 SNPs/haplotypes and responsiveness to treatment. Since the beginning of ADAM33 analysis, researchers have been trying to find an application for their findings in clinical studies. While many initial ideas may not have demonstrated effectiveness, over time, some potential solutions have emerged and will likely continue to develop in the future [32,42,56]. However, the concept of utilizing ADAM33 SNPs as a determinant for therapy responsiveness is still relatively new, and thus, there are limited concrete data available.

Starting with last year’s study of the responsiveness to inhaled corticosteroids and a long-acting β-agonist (ICS + LABA) in asthma, where quite relevant results were obtained from almost a thousand patients (486 controls and 503 cases), the most significant conclusion was initially based on the observation where patients with moderate- and mild-severity asthma did not indicate any improvement, or a very modest one, after a standard procedure of three months of ICS + LABA. Throughout the study, this group was examined in terms of ADAM33 polymorphism. As a result, the homozygous minor allele of SNP V4 (rs2787094) was finally administrated to worsen lung function and to be the causative factor of ICS + LABA therapy failure. With these findings, SNP V4 (rs2787094) could be applied in the future as a dependable genetic marker to define the prospective effect of ICS + LABA therapy and to help limit the time of ineffective therapy. Additionally, in this study, the rest of the examined SNPs, which are considered to be associated with asthma, indicated positive results and significant improvement in the spirometry tests. In particular, rs2853209, described as a protective factor against asthma, indicates the grand improvement [42].

Moreover, another anchor point is the relationship between IgE concentration with asthma profile as well as with ADAM33 polymorphism. This is not as direct as the first finding but is worth mentioning. Knowing which polymorphisms are connected with the growing IgE level, the group of related SNPs can be established. These types are more likely to correspond with the treatments that are aimed at allergic asthma. While IgE tests are generally more efficient, faster, and cost-effective, there may be instances with uncertain outcomes where ADAM33 tests can offer supplementary data to support the maintenance of effective treatment. Looking at the study results, some SNPs are strongly connected with IgE concentrations, e.g., as mentioned earlier, V4, which indicates a strong association with IgE both in adults and children [57].

The interplay between IgE concentration, specific polymorphisms, and responsiveness to particular therapy forms is promising. This clinical chain could be used in the future as one of the diagnostic tools for asthma patients.

### 4.2. Gene Therapy

ADAM33 has been a promising target in research and therapeutic applications in asthma management. A hypothesis emerged that suppressing ADAM33 expression could lead to reduced airway hyperresponsiveness and remodeling [58,59]. Due to the fact that asthma-related ADAM33 SNPs do not belong to the metalloprotease domain, as well as the high level of redundancy within proteases that contribute to asthma pathogenesis through ligand cleavage and ultimately the poor specificity profile of small-molecule metalloproteinase inhibitors, the approach of inhibiting ADAM33′s expression with the pharmacological agent did not fulfill the expectations [58,60]. Therefore, the focus was shifted to a different approach.

Newer and more sophisticated methods involve reducing ADAM33 expression via gene silencing. One study set out to find the most optimal mechanism by which the gene could be silenced. Of four different options, one emerged as the most effective, locked nucleic acid (LNA) gapmer antisense oligonucleotides (ASOs), and was significantly more potent than other variants, especially when transfected with a cationic lipid. Unfortunately, given that a transfecting agent such as Lipofectamine RNAiMAX is rather toxic and not suited for further in vivo research, a process called gymnosis was explored. In this study, gymnotic transfection has shown low micromolar potency and no toxicity compared to the cationic lipid. However, after the 7-day period proposed by the researchers as the most optimal time of observation, the gymnotic transfection showed 60–80% silencing of ADAM33 expression. Therefore, this process has been marked as ideal for further testing [60].

In a recent study, researchers demonstrated, using intratracheal administration of LNA gapmer ASOs into mice lungs, a robust silencing of ADAM33 expression in mice’s fibroblasts. This approach of locally administered ASOs showed good penetration; silencing was observed both in proximal and distal regions of the lung, slightly reduced silencing was evident in the bronchus (75% vs. 55%), and relatively low systemic exposure—over 80% in the bulk lung tissue, less than 50% in the liver, and even lower (30%) in kidneys (in systemic administration, most of the silencing was observed in the liver)—was seen. Furthermore, LNA gapmer ASO managed to robustly silence the gene for a prolonged period of 4 weeks, peaking at 1 week. The study presented novel methods of evaluating these effects by using flow cytometry and more importantly, for the first time, single-cell RNA sequencing (scRNA-seq), which allows studies on oligonucleotide therapeutics in cell types that were inaccessible before. As of writing, there is still more exploring and further research to be carried out for gene silencing methods and oligonucleotide therapeutics; nevertheless, there is great potential for them to be used in future asthma management [61].

## 5. Conclusions

This review paper focuses on the role of the ADAM33 gene in the pathogenesis, susceptibility, severity, and hypothetical treatment of asthma. Although ADAM33′s role in asthma is evident, there is still a need for an in-depth analysis of the significance of particular SNPs among various populations as well as of the exact mechanism in which they exert their impact. Only then will it be possible to apply this knowledge to establish a new asthma therapy targeted towards ADAM33.

## Figures and Tables

**Table 1:** Selected SNPs of significant importance for populations, along with their characteristics.

SNP	Population	Characteristics
F + 1	Asian in general [9], children in general [9], Jordanian [34], Northern Indian [34], German [34], Icelandic [34], and Chinese [34]	Associated with lung function deterioration in early life [34]
Q1,	Asian in general [9], Chinese Han [9], adults in general [9], and Caucasian children [9]	Homozygous for minor alleles of SNP Q-1 (CC), rapid decline in FEV1 of 9.6 mL/year [34]
T2,	Asian in general [9,35], children in general [9], West Bengal, and India [38]	Associated with the type 2 endotype of asthma [20]
S + 1	South India [41]	
S1	West Bengal, India [38]	Interaction between prenatal exposure to cigarette smoke in relation to the development of BHR [43] Associated with respiratory impedance at the age of 8 [43]
S2	Jordanian, British, Europeans, Black Americans, White Americans, Hispanic Americans, and Thai [34,35]	Interaction between prenatal exposure to cigarette smoke in relation to the development of BHR [43] associated with predicted FEV1% [43]
ST + 4	Children in general [9]	
ST + 7	US White and Dutch White [10]	
T1	Asian children [32], West Bengal, India [38], and Iraqi Arab population [14,39]	An allele associated with higher eosinophil count, increased airway hyperresponsiveness [36], increased inflammatory cell counts, and a decline in lung function in patients with COPD [32] Site corresponds to the domain involved in intracellular signaling [32]
V4	Caucasian in general [9], adults in general [9], Jordanian children [34], United States/United Kingdom combined children [34], United Kingdom children [34], Dutch White children [34], Indian children [34], Dutch in general [10], and South India [40]	Associated with an increased risk of asthma with no significant differences in population selectivity [13] Located in the 3′UTR; changes may affect transcription in relationship to closely located T1 [34,35] Linked to decreased lung function and impaired improvement in lung function after three months of ICS + LABA [40] Heterozygotes with reduced FEV1 among smokers have been observed [42]

## Data Availability

No new data were created.
